# Supplementary figures and images for: Phytochemical screening and in vitro antibacterial activity of Echinops kebericho Mesfin tuber extracts: experimental studies
Source: PeerJ. 2024 Dec 3;12:e18554. doi: 10.7717/peerj.18554 (PMC11623108; doi:10.7717/peerj.18554)

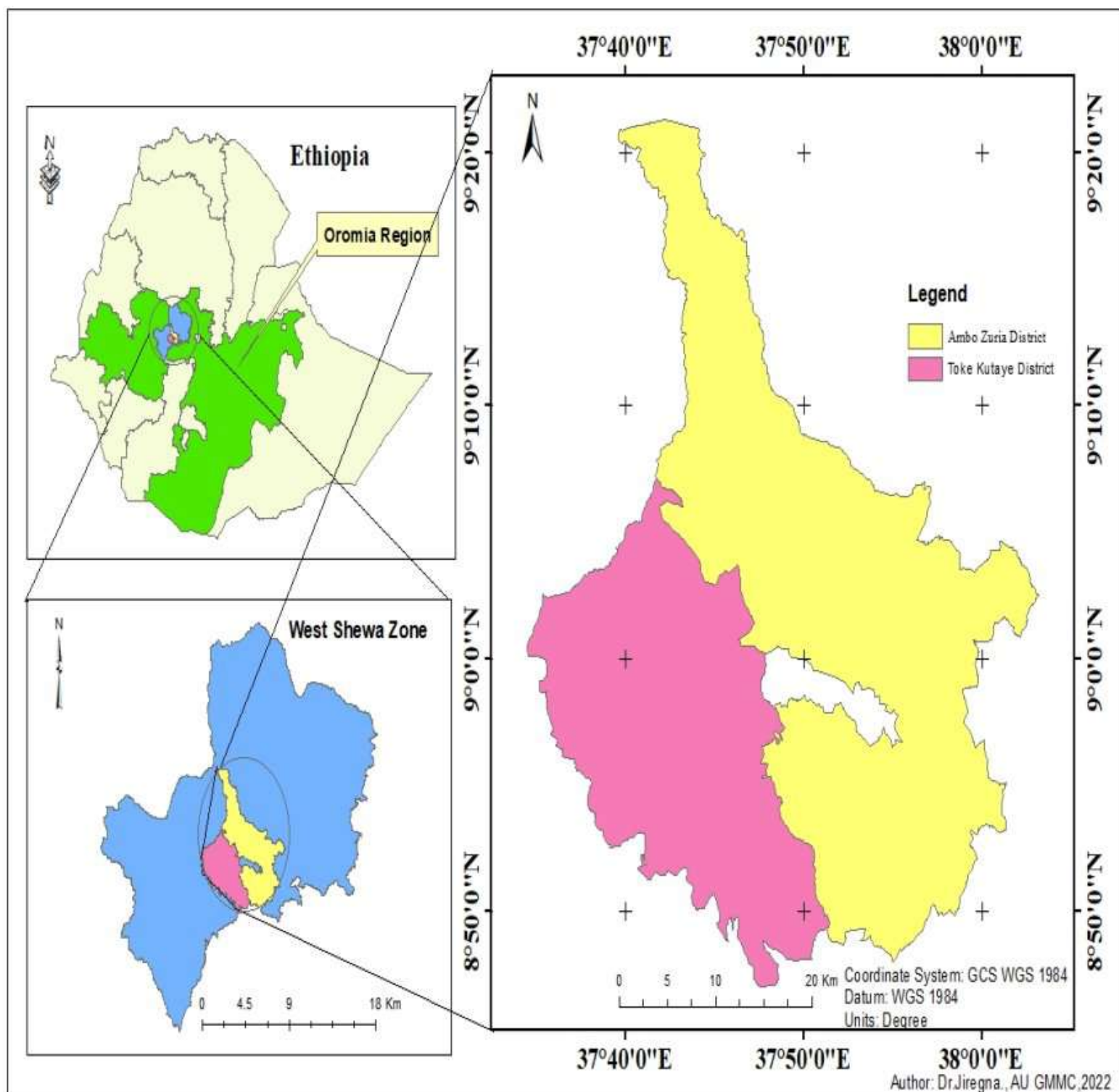

Supplement: Supplemental Information 3 — Source: Adopted from Arc GIS Computation and Ethiopian Map, 2021. [file peerj-12-18554-s003.pdf]
